# Circulating Endothelial Progenitor Cell and Platelet Microparticle Impact on Platelet Activation in Hypertension Associated with Hypercholesterolemia

**DOI:** 10.1371/journal.pone.0052058

**Published:** 2013-01-25

**Authors:** Nicoleta Alexandru, Doina Popov, Emanuel Dragan, Eugen Andrei, Adriana Georgescu

**Affiliations:** 1 Petru Poni’ Institute of Macromolecular Chemistry, Iasi, Romania; 2 Institute of Cellular Biology and Pathology, ‘Nicolae Simionescu’ of the Romanian Academy, Bucharest, Romania; National Cancer Institute, United States of America

## Abstract

**Aim:**

The purpose of this project was to evaluate the influence of circulating endothelial progenitor cells (EPCs) and platelet microparticles (PMPs) on blood platelet function in experimental hypertension associated with hypercholesterolemia.

**Methods:**

Golden Syrian hamsters were divided in six groups: (i) control, C; (ii) hypertensive-hypercholesterolemic, HH; (iii) ‘prevention’, HHin-EPCs, HH animals fed a HH diet and treated with EPCs; (iv) ‘regression’, HHfin-EPCs, HH treated with EPCs after HH feeding; (v) HH treated with PMPs, HH-PMPs, and (vi) HH treated with EPCs and PMPs, HH-EPCs-PMPs.

**Results:**

Compared to HH group, the platelets from HHin-EPCs and HHfin-EPCs groups showed a reduction of: (i) activation, reflected by decreased integrin 3β, FAK, PI3K, src protein expression; (ii) secreted molecules as: SDF-1, MCP-1, RANTES, VEGF, PF4, PDGF and (iii) expression of pro-inflammatory molecules as: SDF-1, MCP-1, RANTES, IL-6, IL-1β; TFPI secretion was increased. Compared to HH group, platelets of HH-PMPs group showed increased activation, molecules release and proteins expression. Compared to HH-PMPs group the combination EPCs with PMPs treatment induced a decrease of all investigated platelet molecules, however not comparable with that recorded when EPC individual treatment was applied.

**Conclusion:**

EPCs have the ability to reduce platelet activation and to modulate their pro-inflammatory and anti-thrombogenic properties in hypertension associated with hypercholesterolemia. Although, PMPs have several beneficial effects in combination with EPCs, these did not improve the EPC effects. These findings reveal a new biological role of circulating EPCs in platelet function regulation, and may contribute to understand their cross talk, and the mechanisms of atherosclerosis.

## Introduction

Cardiovascular diseases, the leading cause of morbidity and mortality in industrialized countries are predominantly caused by atherosclerosis. This is an inflammatory disease, the result of a cascade of events in blood vessels, leading to remodelling of the arterial wall, and a subsequent reduction in lumen size.

In recent years, traditional risk factors for atherosclerosis (such as hypertension and hypercholesterolemia) have also been associated with decreased numbers and impaired function of circulating endothelial progenitor cells (EPCs) [1], [2], [3], [4], [5]. EPCs released by bone marrow, fat tissue and the vessel wall (especially adventitia), and possibly spleen, liver, and intestine, into blood, express CD133 at the early stage, and then CD34/Flk-1 [6]. Currently, many studies have shown two important functions of EPCs in the cardiovascular system: regeneration of the endothelial layer and formation of new blood vessels [7]. The discovery of EPCs opened the way for studies on vascular regeneration, and a new perspective in these cell-based methods [7]. It was demonstrated that EPCs generated in vitro from peripheral blood mononuclear cells (PBMCs) have potential therapeutic applications in vascular tissue engineering and cell-based methods [8], [9], [10], [11]. Newly, has been demonstrated that bio-engineered EPCs-capture stent technology is successful in EPCs capture in the human circulation decreasing thrombogenicity [12].

EPC recruitment towards vascular lesions, a critical step in atherosclerosis, is mediated by their interaction with platelets, the blood cells patrolling the vascular wall for endothelial integrity [13], [14], [15], [16]. Platelets are involved in EPC homing by releasing potent chemotactic factors such as the stromal-cell-derived factor-1 (SDF-1α). In an in vitro model it was shown that a significant interaction occurs between activated platelets and EPCs under both static and flow conditions, and this interaction is mediated by P-selectin–P-selectin glycoprotein ligand-1 (PSGL-1) binding and β1- and β2 integrins [17], [18], [19], [20]. Moreover, in vivo experiments of carotid injury in mice have reported that platelets provide a critical signal for the early recruitment of bone marrow (BM) -derived progenitor cells, such as CD34+ cells, to the sites of vascular injury [21]. Apart from EPC homing to sites of vascular injury, platelets support and promote the maturation and differentiation of EPCs to endothelial cells (ECs) [18], [19], [22]. Furthermore, it has shown that activated platelets induce differentiation of CD34^+^ progenitor cells into ECs and macrophages/foam cells [19], [23]. So, an altered balance of platelet-mediated transformation of CD34^+^ progenitor cells plays a critical role in atherogenesis and atheroprogression. Conversely, EPCs may influence platelet function and modulate their thrombogenic properties during vascular repair. EPCs secrete many vasoactive and angiogenic factors that may modulate vascular thrombosis and hemostasis. In one study it was shown that human PBMC-derived EPCs bound platelets via P-selectin and inhibit platelet activation, aggregation, adhesion to collagen, and thrombus formation, predominantly via upregulation of cyclooxygenase-2 and secretion of prostacyclin [24].

Recently, it was suggested that the ratio between EPCs and microparticles (MPs), small membrane vesicles endowed with pro-coagulant and pro-inflammatory properties, may be considered a marker of vascular dysfunction [5]. Platelet microparticles (PMPs) are the most abundant microparticles in the bloodstream constituting approximately 70% to 90% of circulating microparticles [25]. Elevated PMPs levels are found in the blood of patients with diseases associated with thrombosis, for example: heparin-induced thrombocytopenia, arterial thrombosis, idiopathic thrombocytopenic purpura, thrombotic thrombocytopenia, sickle cell disease, uremia, chronic venous insufficiency, hypertension associated with hypercholesterolemia [5], [25], [26].

The present study was designed to provide new insight into EPCs-based therapy in atherosclerosis, and to assess the role of PMPs, alone and in correlation with EPCs, on platelet functions in the experimental model of hypertension-hypercholesterolemia, reported previously by our group [5]. Here we describe new approaches towards pathology amelioration, HH hamsters treated with PBMC-derived EPCs for prevention (HHin-EPCs), HH treated with PBMC-derived EPCs for regression (HHfin-EPCs), HH treated with PMPs (HH-PMPs) and HH treated with EPCs and PMPs (HH-EPCs-PMPs).

## Methods

### Experimental Models

The experiments were performed on platelets isolated from the blood of golden Syrian hamsters (3 months of age, n = 120) divided in six groups: (i) control, C (fed a standard hamster diet); (ii) hypertensive-hypercholesterolemic, HH (fed standard diet enriched with 3% cholesterol, 15% butter and 8% NaCl, for 4 months as described by Alexandru et al. [27] and Georgescu et al. [5]; (iii) HH treated with EPCs (as prevention group), HHin-EPCs [fed as HH group for 4 months and injected via the retro-orbital plexus with 1×10^5^ EPCs (isolated from C group) in one dose per month during diet-induced atherosclerotic process] [28]; (iv) HH treated with EPCs after induction of hypertension and hypercholesterolemia (as regression group), HHfin-EPCs [fed as HH group for 4 months and then injected via the retro-orbital plexus with 1×10^5^ EPCs (isolated from C group) in one dose per month during the other 4 months; (v) HH treated with PMPs, HH-PMPs, [fed as HH group for 4 months and injected via the retro-orbital plexus with 1×10^5^ PMPs (isolated from HH group) in one dose per month during diet-induced atherosclerotic process] and (vi) HH treated with EPCs and PMPs, HH-EPCs-PMPs, [fed as HH group and injected via the retro-orbital plexus with 1×10^5^ EPC (isolated from C group) and 1×10^5^ PMPs (isolated from HH group) in one dose per month during the 4 months of diet].

For all groups of animals, the systolic and diastolic arterial blood pressure were recorded using a Physiological Pressure Transducer (model MLT844/D) connected to the PowerLab data acquisition unit (ADInstruments, Sydney, Australia). The plasma cholesterol and triglyceride concentrations were assayed by the use of: (i) assay strips, monitoring the values with the Accutrend GCT (Roche, USA) apparatus; and (ii) enzymatic kits (Sigma Chemical Co., MO, USA). For the characterization of experimental animal models, the above parameters, as well as the body weight were recorded both at the start and the end of the 4 months experiment.

Experiments on animals were conformed to the Guide for the Care and Use of Laboratory Animals published by the US National Institutes of Health (NIH Publication no. 85–23, revised 1985) and were approved by the Ethics Committee of ICBP ‘N. Simionescu’.

### Platelet Isolation

Hamsters were slightly ether anesthetized, and blood was collected from the retro-orbital plexus. Platelets were separated according to the method reported by Lupu et al. [29] and Alexandru et al. [27]. Briefly, the procedure consists in collection of venous blood in ACD buffer (2.73% citric acid, 4.48% trisodium citrate and 2% glucose) and centrifugation at 400×g for 10 min. PRP obtained was spun down at 600×g for 10 min, and the platelets suspended in calcium-free HEPES buffer (pH 7.0) supplemented with 1% BSA and 0.15 U/ml apyrase. Phase contrast microscopy of the pellet showed that these were not aggregated, and the preparation was devoid of erythrocytes and leukocytes.

### EPC Isolation

PBMCs were fractionated using HISTOPAQUE-1077 density-gradient centrifugation (400 g 30 min, at 24°C) as described by Georgescu et al. [5]. The mononuclear cells were isolated, washed with phosphate-buffered saline (PBS) supplemented with 2% fetal serum and finally, resuspended in PBS supplemented with 2% fetal serum. EPCs were sorted from PBMCs using the specific antibody for VEGFR2, CD34 by flow cytometry and were adjusted at the same number of 1×10^5^/ml in PBS.

### Preparation of Platelet-free Plasma (PFP), the Source for Circulating PMPs

Plasma PMPs were separated according to the method reported by Georgescu et al. [5], [26]. Briefly, the procedure consists in collection of venous blood in 0.138 M tri–sodium citrate 9/1 (vol/vol), centrifugation at 1000×*g* for 15 min, at 15°C and separation of platelet rich plasma (PRP). PRP was centrifuged at 2500×*g* for 15 min, at 15°C and the PFP obtained was centrifuged again at 13 000×*g* for 5 min at 15°C allowing collection of PMPs in the supernatant.

### PMP Sorting

PMPs were sorted from PFP according to Georgescu et al. [5], [26] using specific antibodies to integrin αIIb (M 148) PE (for CD41) and annexin V FITC (for PS) and the MoFlo flow cytometer (Dako, USA) equipped with high-speed cell sorter. The number of PMPs was adjusted at 1×10^5^/ml in PBS.

### Flow Cytometry

Freshly isolated platelets were suspended in modified Tyrodes-HEPES buffer containing 0.35% BSA according to the method reported by Bergmeier et al. [30] and Alexandru et al. [27]. Suspensions containing the same number of platelets, (i.e 5×10^5^ cells) were incubated with antibody to integrin beta 3 (Phycoerythrin) at dark and room temperature, for 30 min. The reaction was stopped by adding paraformaldehyde (PFA) (final concentration 1%), and samples were analyzed for 10 000 events each with the MoFlo flow cytometer (Dako,USA).

### Platelet Supernatant Preparation

Platelet supernatant was obtained according to the method described by Dernbach et al. [4]. Isolated platelets as above were resuspended in HEPES buffer (∼1×10^6^ platelets/mL) and were activated by centrifugation at 10.000×g, for 10 min. The supernatant thus obtained was used for cytokine/chemokines assay.

### SDS-PAGE and Immunoblotting Detection of Platelet Proteins

Platelet protein analysis was performed as previously reported [27]. The intensity of protein bands on blots was evaluated using TL100 1D computer program from Nonlinear Dynamics, (Newcastle upon Tyne, UK).

### Cytokine/Chemokines and Growth Factors Assays

Platelet supernatants isolated as described above were used to measure the concentration of the following molecules: SDF-1, monocyte chemotactic protein-1 (MCP-1), Regulated upon Activation, Normal T-cell Expressed, and Secreted (RANTES), vascular endothelial growth factor (VEGF), platelet factor 4 (PF4), platelet-derived growth factor (PDGF), tissue factor pathway inhibitor (TFPI) by enzyme-linked immunosorbent assay (ELISA, R&D Systems, Wiesbaden) using the specific kits according to the manufacturer’s instructions. Briefly, samples were added in each well of a 96-well microtiter plate coated with antibody anti-specific ckemokine, and incubated for 1 or 2 hours at room temperature. After washing, adding of conjugate, substrate and stop solution, the optical density at 450 nm was measured using a spectrophotometer (TECAN, InfiniteM200PRO, Austria).

### Reagents

The standard chemicals, reagents and the specific antibodies were purchased from Sigma Chemical Co. (St. Louis, MO, USA), Santa Cruz Biotechonolgy (www.scbt.com USA), and from Abcam (http://www.abcam.com, USA). All others reagents used were of analytical grade.

### Data Analysis

Statistical evaluation of the results was performed using One-Way Analysis of Variance (Anova) and the Student t-test; data were considered statistically significant when *p*≤0.05.

For the flow cytometry experiments a software based on auto and manual compensation was used (SUMMIT 4.0 b2060 Software, DakoCytomation, USA).

## Results

### Assessment of Biochemical Parameters and of Hypertension in the Animal Model

Compared to normal hamsters in group C that displayed values of cholesterol and triglyceride concentrations (154.55±8.74 mg/dL and 217.66±24.98 mg/dL), systolic and diastolic blood pressure (89.24±1.58 mm Hg and 60.25±2.88 mm Hg) characteristic for the species, the group HH displayed 825.53±84.23 mg/dL cholesterol, 419.75±33.60 mg/dL triglyceride, 137.89±1.89 mm Hg systolic blood pressure, and 107.14±4.53 mm Hg diastolic blood pressure. The HHin-EPCs and HHfin-EPCs groups showed 650.34±29.82 mg/dL and 179.75±9.33 mg/dL cholesterol, 368.22±37.67 mg/dL and 286.28±17.76 mg/dL triglyceride, 119.77±1.63 mm Hg and 101.55±1.35 mm Hg systolic blood pressure and 95.12±4.33 mm Hg and 84.57±3.86 mm Hg diastolic blood pressure. These results indicate that EPC treatment was efficient in reducing these parameters. The HH-PMPs group was characterized by 1132.14±119.40 mg/dL for cholesterol, 502.33±26.41 mg/dL for triglyceride, 159.78±2.07 mm Hg for systolic, and 128.39±5.16 mm Hg for diastolic blood pressure. The HH-EPCs-PMPs group had: 904.12±35.52 mg/dL for cholesterol, 406.88±46.87 mg/dL for triglyceride, 95.57±1.23 mm Hg for systolic blood pressure and 71.69±3.77 mm Hg for diastolic blood pressure. Thus, the combined EPCs with PMP administration had a beneficial effect on blood pressure, reducing their values vs. those in HH group, except for cholesterol and triglyceride concentrations. In time, the body weight of the investigated animal groups was slightly increased, the values being in the same range ∼111.25±9.55 g both at the start and at the end of the 4 months experiment. Compared to C group, the values for glucose concentration were not significantly different between the investigated animal models.

### Assessment of Integrin β 3 Expression

The activation of platelets results in a rapid series of a variety of signal transduction events, including the conformational change of α_IIb_β_3_, with exposure of its high-affinity ligand binding site. Flow cytomety analysis showed that the percent of events marked by the antibody to integrin-β3-PE was increased for platelets isolated from HH, HH-PMPs and HH-EPCs-PMPs groups by ∼3.02-fold, ∼4.37-fold and by ∼3.55- fold, respectively, compared to C group (Table 1 and Fig. 1.1,1.2,1.5,1.6) (*p*≤0.001). Moreover, the percent of events marked by the above antibody were increased by ∼1.57-fold and ∼1.45-fold, in the platelets from HHin-EPCs and HHfin-EPCs groups, respectively, compared to the percent recorded in platelets from C group, and were reduced by ∼1.93-fold, ∼2.08-fold compared to the percent recorded in platelets from HH group (Table 1 and Fig. 1.3, 1.4) (*p*≤0.01).

**Figure 1:**
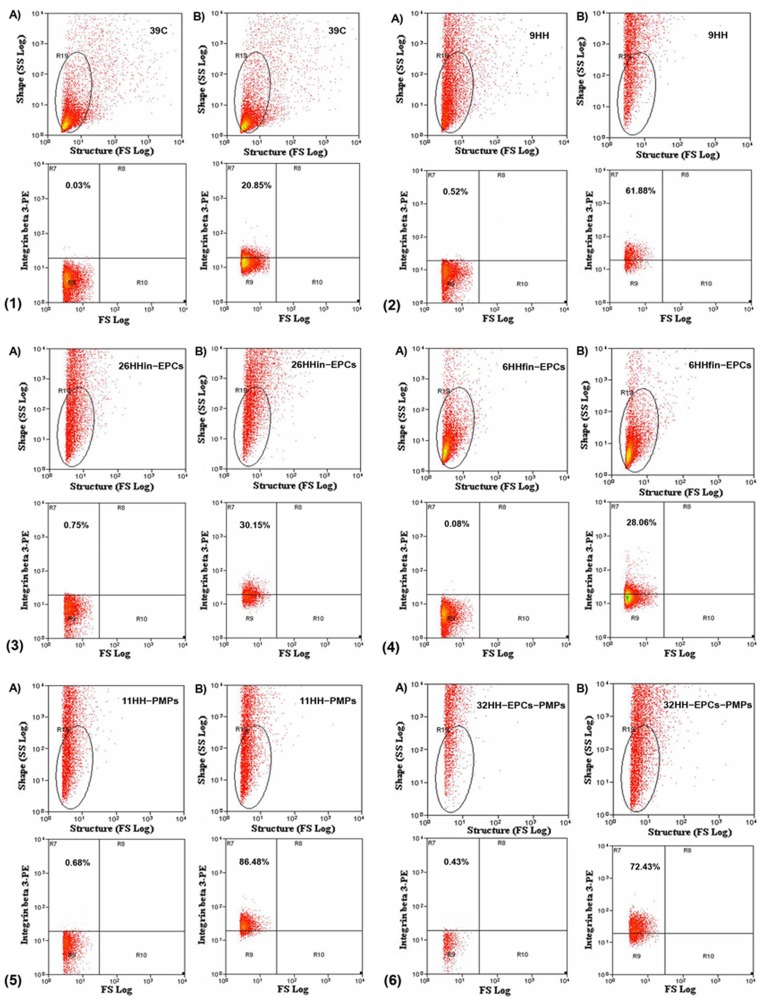
The flow cytometric detection on platelet activated Integrin β- 3 (1): control group, C (2): hypertensive- hypercholesterolemic (HH) group; (3): prevention group, HHin-EPCs (4) regression group, HHfin-EPCs (5) HH treated with PMPs group, HH-PMPs and (6) HH treated with EPCs and PMPs, HH-EPCs-PMPs. The left panel (A): representative unmarked sample; the right panel (B): representative sample marked with Integrin β3 antibody. The marked events for Integrin β3 are illustrated in gates R7.

**Table 1:** Integrin β3 expression on the platelet membrane.

Hamster groups	Percent of events marked for Integrin beta 3-PE (%)
**Control (n = 10)**	20.545±1.4582
**HH (n = 8)**	62.06±3.1520 (*p≤0.001)
**HHin-EPCs (n = 7)**	32.19±1.04 (**p≤0.01)
**HHfin-EPCs (n = 9)**	29.833±2.8960 (**p≤0.01)
**HH- PMPs (n = 7)**	89.773±2.379 (*p≤0.001, **p≤0.001)
**HH-EPCs-PMPs (n = 8)**	72.913±5.302 (*p≤0.001)

Data are means ± SEM. The statistical significance, noticeably different was represented as *p values (for comparisons with C group) and as **p (for comparisons with HH group). n = the number of animals.

Thus, compared to HH hamsters, EPC treatment (in both situations, prevention/regression) reduces exposure of integrin β3 on platelet membrane. The simultaneous administration of EPCs and PMPs reduces the exposure of integrin β3 on platelet membrane comparative vs. HH-PMPs group, and is not more efficient than EPC treatment, only.

### Evaluation of Signaling Pathways Involved in Platelet Aggregation

In order to uncover the platelet hyperaggregability, we investigated the molecules involved in α_IIb_β_3_ integrin signalling, such as FAK, Src, and p85 subunit of PI3-Kinase in platelets isolated from the experimental groups.

**Figure 2:**
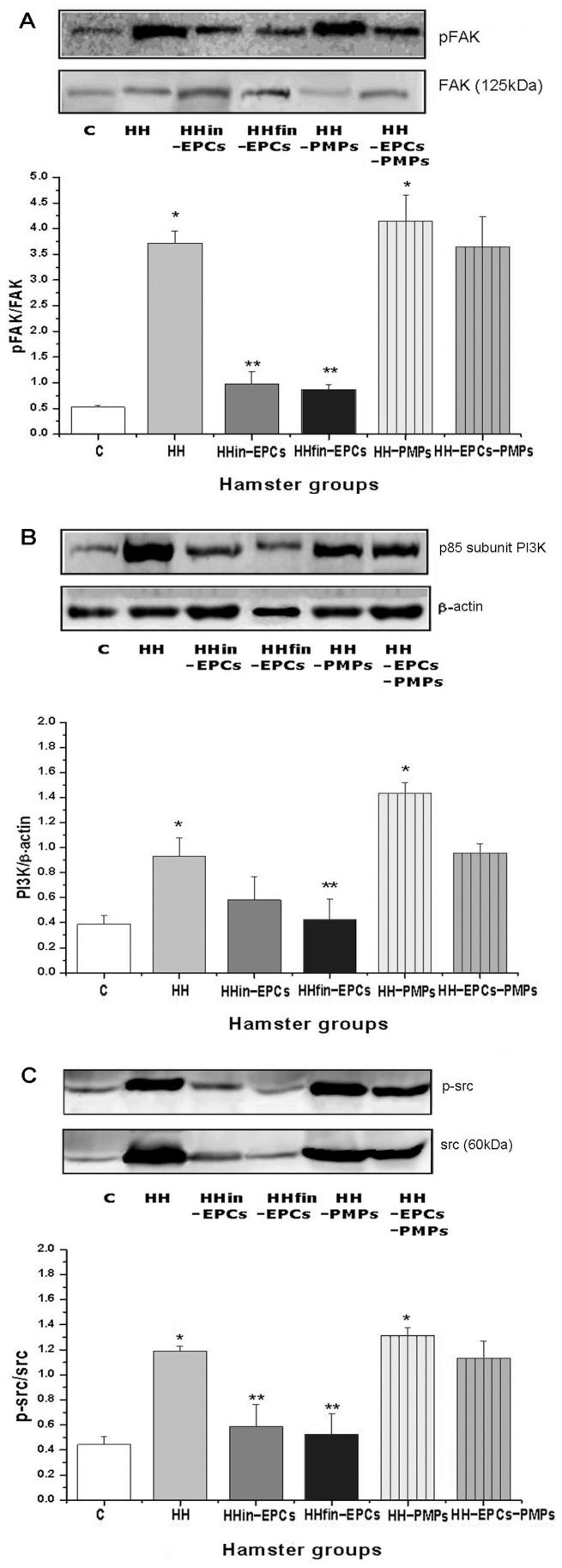
Representative immunoblots and densitometric data for the platelets from hamster groups: control (C), hypertensive-hypercholesterolemic (HH), prevention (HHin-EPCs), regression (HHfin-EPCs), HH treated with PMPs (HH-PMPs) and HH treated with EPCs and PMPs (HH-EPCs-PMPs). (A): pFAK, FAK, (B): p85 subunit of PI3K, β- actin, (C): p-src, src. (*) Groups vs Control: *p*≤0.05. (**) Groups vs HH: *p*≤0.05.

Compared to C group, the densitometric analysis of immunoblots presented that the pFAK/FAK ratio was increased by ∼7.1-fold at HH group, ∼1.88-fold at HHin-EPCs, ∼1.66-fold at HHfin-EPCs, ∼7.95-fold at HH-PMPs and ∼6.98-fold at the platelets isolated from HH-EPCs-PMPs group (n = 4, Fig. 2A). Compared to HH group, in HHin-EPCs and HHfin-EPCs groups, the values for pFAK/FAK ratio were reduced by ∼3.78-fold, and ∼4.3-fold respectively (*p*≤0.05). Compared to C group, the protein expression of PI3K was higher by ∼2.4-fold in HH group, ∼1.5-fold in HHin-EPCs, ∼1.1-fold in HHfin-EPCs, ∼3.7-fold in HH-PMPs and ∼2.46-fold in HH-EPCs-PMPs group (n = 4, Fig. 2B). Compared to HH group, in HHin-EPCs, and HHfin-EPCs the values for PI3K were reduced by ∼1.6-fold, and by ∼2.19-fold, respectively (*p*≤0.05). The Western blotting experiments for src showed similar results, with a significant raise in its expression in HH and HH-PMPs groups, vs. C group. Thus, the increase in p-src/src ratio was by ∼2.68-fold in platelets isolated from HH group, and by ∼2.96-fold in platelets isolated from HH-PMPs group (n = 4, Fig. 2C); the augmentation measured ∼1.33-fold in HHin-EPCs group, ∼1.19-fold in HHfin-EPCs and ∼2.56-fold in platelets isolated from HH-EPCs-PMPs group (n = 6, Fig. 2C). Compared to HH group, in HHin-EPCs and HHfin-EPCs groups, the values for p-src/src ratio were reduced by ∼2.02-fold and by ∼2.25-fold, respectively (*p*≤0.05). Moreover, compared to HH group, the value for pFAK/FAK, PI3K and p-src/src ratio were augmented by ∼1.12-fold, ∼1.54-fold, and ∼1.1-fold in platelets isolated from HH-PMPs group, and were not significantly changed in platelets from HH-EPCs-PMPs group.

Taken together, these data demonstrate that EPC treatment (both in prevention and in regression situation) modulates the platelet signaling protein expression, and reduces their activation towards the values recorded in controls. The levels of analyzed proteins recorded in the HH-PMPs group were significantly enhanced (*p*≤0.05), compared to C group; administration of EPCs together with PMPs reduces the values compared to HH-PMPs group, but is not so efficient as EPC administration, only.

### Evaluation of Cytokine/Chemokines and Growth Factors in Supernatants of Activated Platelets

The activation of platelets results in the release of various cytokines, which might be able to exert putative effects on EPC functions in a paracrine manner. Therefore, we measured the concentration of several cytokine/chemokines and growth factors in the supernatant of platelets activated by centrifugation.

Because SDF-1 is a platelet-derived factor involved in EPC recruitment, we first assayed the levels of SDF-1 in the supernatants obtained from hamster group-derived platelets. Compared to C group, the level of SDF-1 was higher especially in platelet supernatant isolated from HH, HH-PMPs groups; the enhancement was by ∼1.15-fold and ∼1.20-fold, respectively for these groups (Table 2). The concentrations of this chemokine for HHin-EPCs, HHfin-EPCs and HH-EPCs-PMPs groups were similar to the value for C group. Compared to HH group, the decrease was by ∼1.18-fold for HHin-EPCs, ∼1.09-fold for HHfin-EPCs and ∼1.18-fold for HH-EPCs-PMPs, respectively; in platelet supernatant from HH-PMPs group the value was insignificantly modified.

**Table 2:** Chemokine and factor release in platelet supernatants of investigated hamster groups (n = 6 for all molecules; concentration expressed as pg/mL).

	C	HH	HHin- EPCs	HHfin-EPCs	HH-PMPs	HH-EPCs-PMPs
**SDF-1**	270.333±4.24	312±5.851	263.7±6.703	286.25±3.038	323.5±9.946	263.875±7.06
**RANTES**	2.051±0.785	4.012±0.245	2.679±0.518	2.391±0.177	3.257±0.266	2.622±0.681
**MCP-1**	99.331±6.563	220.852±48.494	103.746±8.199	85.657±12.211	336.215±32.372	324.291±43.584
**PF4**	1 645.341±647.022	3 331.41±259. 459	1 924.776±563.288	1 415.775±232. 942	3 509.817±235.674	1 715.476±392.568
**VEGF**	14.264±0.408	17.924±2.857	12.668±0.769	13.366±0.999	19.989±0.603	15.419±0.329
**PDGF-AB**	5.356±0.814	7.845±0.712	5.350±1.754	5.559±1.168	9.285±0.765	8.910±2.929
**TFPI**	87.306±2.475	68.169±3.806	103.507±6.788	96.043±7.022	62.898±4.319	77.370±1.518

Next, we measured the concentration of platelet chemokines RANTES and MCP-1, known to be involved in inflammation, atherogenesis, and vascular remodeling after injury [31]. Thus, compared to C group, RANTES concentration was enhanced in platelet supernatant, in the range: ∼1.96-fold for HH, ∼1.31-fold for HHin-EPCs, ∼1.17-fold for HHfin-EPCs, ∼1.59-fold for HH-PMPs, and ∼1.28-fold for HH-EPCs-PMPs (Table 2). Compared to HH group, all other experimental groups displayed reduced levels of RANTES, as follows: ∼1.50-fold for HHin-EPCs, ∼1.68-fold for HHfin-EPCs, ∼1.23-fold for HH-PMPs, ∼1.53-fold for HH-EPCs-PMPs.

Measurement of MCP-1 concentration in platelet supernatants isolated from HH, HH-PMPs and HH-EPCs-PMPs groups revealed a significantly augmentation, compared to C group, of ∼2.22-fold, ∼3.38-fold and ∼3.26-fold, respectively (Table 2). In the samples from HHin-EPCs and HHfin-EPCs, MCP-1 concentration was comparable to C group. Moreover, compared to HH group we recorded decreased values in platelet supernatant from these groups by: ∼2.13-times for HHin-EPCs and ∼2.58-times for HHfin-EPCs, respectively. As showed in Table 2, MCP-1 levels were higher not only in platelet supernatant generated from HH-PMPs group (of ∼1.52-times), but also in sample from HH-EPCs-PMPs (of ∼1.47-times), compared to HH group (Table 2).

Platelet factor 4 (PF4) released by platelets is delivered, like RANTES, to the monocyte and endothelium surface, respectively, where induces the activation of monocyte-related integrins, and eventually promotes macrophage infiltration in the vascular wall [32]. Compared to C group, PF4 concentration in platelet supernatant, was higher for almost all animal groups: HH (∼2.02-fold), HHin-EPCs (∼1.17-fold), and HH-PMPs (∼2.13-fold); in HH-EPCs-PMPs group the value was insignificantly modified. Compared to HH group, we recorded decreased values for platelet supernatant by ∼1.73-fold in HHin-EPCs group, ∼2.35-fold in HHfin-EPCs group, and ∼1.94-fold in HH-EPCs-PMPs group; in HH-PMPs group the PF4 concentration was slightly enhanced.

In the following experiments we assayed the presence of pro-angiogenetic factors, VEGF and PDGF-AB in platelet supernatants. The results show that compared to C group, the values for VEGF were increased by ∼1.26-fold in HH group, ∼1.40-fold in HH-PMPs group and ∼1.08-fold in HH-EPCs-PMPs group. The concentrations measured in HHin-EPCs and HHfin-EPCs groups were similar to de value in C group. Compared to HH group, in HHin-EPCs, HHfin-EPCs and HH-EPCs-PMPs groups, the values for VEGF were reduced by ∼1.42-fold, ∼1.34-fold and ∼1.16-fold. In platelets isolated from HH+PMPs group the value for VEGF was enhanced by ∼1.12-fold comparative with HH group (Table 2).

Compared to C group, the analysis of PDGF-AB concentration in platelet supernatants isolated from HH, HH-PMPs and HH-EPCs-PMPs groups revealed an augmentation of: ∼1.46-fold, ∼1.73-fold and ∼1.66-fold, respectively (Table 2). In samples from HHin-EPCs and HHfin-EPCs, PDGF-AB values were comparable to these in C group. Compared to HH group, the values for platelet supernatant from these groups we reduced by ∼1.47-fold in HHin-EPCs and ∼1.41-fold in HHfin-EPCs. Conversely, the platelet supernatants in HH-PMPs and HH-EPCs-PMPs groups displayed a slightly increase of PDGF-AB concentration by ∼1.18-fold and ∼1.14-fold respectively (Table 2).

Platelets are the primary hematopoietic cell accumulating within a growing thrombus, where they release the TFPI, which is the main physiologic inhibitor of tissue factor, the initiator of blood coagulation. Measurement of TFPI concentration in platelet supernatants isolated from hamster groups showed a reduction compared to C group, of ∼1.28-fold (for HH), ∼1.39-fold (for HH-PMPs) and ∼1.13-fold (for HH-EPCs-PMPs) (Table 2). Compared to C group, TFPI in HHin-EPCs and HHfin-EPCs groups is slightly increased i.e. ∼1.19-fold, and ∼1.10-fold, respectively. Compared to HH group, the enhancement observed in HHin-EPCs, HHfin-EPCs and HH-EPCs-PMPs group, was of ∼1.52-fold, ∼1.41-fold and ∼1.135-fold, respectively. The platelet supernatant isolated from HH-PMPs group showed a slight decrease in TFPI of ∼1.08-fold, compared to HH group.

The above results indicate that EPC administration in hypertension associated with hypercholesterolemia reduces the levels of pro-inflammatory molecules secreted by activated platelets and improves the amount of TFPI released by platelets. Moreover, PMP administration induces a general augmentation of secreted molecules, except for TFPI level, that is diminished.

### Estimation of Cytokine/Chemokines Protein Expression

**Figure 3:**
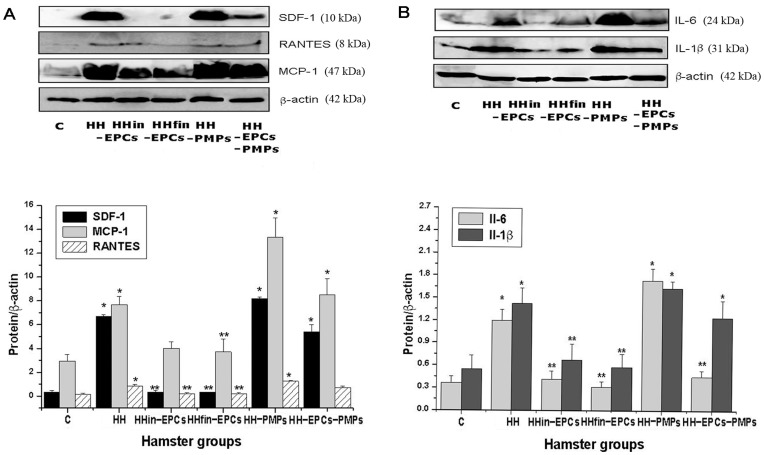
Representative immunoblots and quantification by densitometric analysis for pro-inflammatory molecules released by platelets isolated from the hamsters groups: control (C), hypertensive-hypercholesterolemic (HH), prevention (HHin-EPCs), regression (HHfin-EPCs), HH treated with PMPs (HH-PMPs) and HH treated with EPCs and PMPs (HH-EPCs-PMPs). (A): SDF-1, RANTES, MCP-1, β- actin, (B): Il-6, Il-1β, β- actin. (*) Groups vs Control: *p*≤0.05. (**) Groups vs HH: *p*≤0.05.

The results of immunoblotting experiments revealed that compared to C group (n = 6), in platelets isolated from HH group, protein expressions for SDF-1 and MCP-1 were increased by ∼19.31-fold (n = 4) and ∼2.59-fold, respectively (n = 8) (Fig. 3A). Compared to C group, in platelets isolated from the HHin-EPCs and HHfin-EPCs groups, SDF-1 expressions (n = 4) were unchanged, while MCP-1 expressions (n = 6) were slightly increased by ∼1.36-fold, and −1.26-fold, respectively (Fig. 3A). Compared to HH group, both chemokine expressions were decreased by ∼19.06-fold, ∼20.92-fold and ∼1.91-fold, 2.06-fold. As shown in Fig 3A, compared to the values in C group, the levels of SDF-1 and MCP-1 protein expressions were significantly increased in platelets isolated from de HH-PMPs and HH-EPCs-PMPs groups: by ∼23.71-fold, ∼15.63-fold (n = 4, for SDF-1) and ∼4.51-fold, ∼2.88 fold (n = 6, for MCP-1), respectively (*p≤* 0.05). Compared to HH group, for SDF-1 we obtained an increase of ∼1.23-fold in HH-PMPs group and a reduction of ∼1.24-fold, in HH-EPCs-PMPs, respectively (Fig. 3A). The MCP-1 values in these groups were augmented compared to HH group: ∼1.74-fold in HH-PMPs and ∼1.11-fold in HH-EPCs-PMPs.

Protein expression of RANTES was increased in HH group (∼6.78-fold), in HH-PMPs (∼10-fold) and in HH-EPCs-PMPs group (∼5.8-fold), compared to C group (n = 4, Fig. 3A). The values for HHin-EPCs and HHfin-EPCs were similar to those for C group. Comparative with HH group, for these groups we find a reduction of ∼4.4-fold and ∼4.28-fold, respectively. In platelets isolated from HH-PMPs group the RANTES expression was higher by ∼1.48-fold, while in platelets from HH-EPCs-PMPs its expression was decreased by ∼1.17-fold, compared to HH group.

Next, we evaluated the protein expression of IL-6, which is known to be released from platelets and to induce the expression of the VEGF and exert a pro-angiogenetic effect on endothelial cells (Dernbach 2008). Compared to C group (n = 6), protein expression for Il-6 was increased in platelets isolated from HH group (n = 6) of ∼3.29-times, ∼1.14-times in HHin-EPCs group, ∼4.76- times (n = 5) in HH-PMPs group (n = 5), and ∼1.24-times in HH-EPCs-PMPs group (n = 6); in HHfin-EPCs group the value was slightly decreased of ∼1.16-times (Fig. 3B). Compared to HH group we obtained augmented values for samples from HH-PMPs group by ∼1.45-fold, and reduced ratio for platelets isolated from HHin-EPCs, HHfin-EPCs and HH-EPCs-PMPs groups by: ∼2.88-fold, ∼3.81-fold, and ∼2.66-fold, respectively.

IL-1β is a platelet derived cytokine that has been postulated to be a main mediator of platelet-induced endothelial activation. This cytokine stimulates the endothelium and promotes the activation of endothelial nuclear factor-B, that in turn, triggers the transduction and translation of key genes such as MCP-1, a_v_ß_3_, ICAM-1 and VCAM-1– crucial for monocyte attachment and transmigration [32]. Compared to C group (n = 4), for Il-1β expression our results showed an increase by ∼2.61-fold in HH group (n = 4), ∼1.23-fold in HHin-EPCs group (n = 4), ∼1.06-fold in HHfin-EPCs group (n = 4), ∼3-fold in HH-PMPs group (n = 4), and by ∼2.27-fold in HH-EPCs-PMPs group (n = 4) (Fig. 3B). Compared to HH group, the values were increased by ∼1.15-fold in HH-PMPs group, and diminished by ∼2.13-fold in HHin-EPCs, ∼2.46-fold in HHfin-EPCs group, ∼1.15-fold in HH-EPCs-PMPs group, respectively.

## Discussion

EPCs play a critical role in maintaining endothelial function as well as in progression of cardiovascular disease [33]. At present, only indirect evidence exists for the prevailing understanding that circulating EPCs provide protection against atherosclerosis by their innate ability to replace dysfunctional ECs and to regenerate senescent and damaged endothelium [8], [34]. In a mouse model of vein graft atherosclerosis has been reported endothelial repair by BM-derived EPCs [35] and in hypercholesterolemic (ApoE^−/−^) mice transplanted BM–derived EPCs were found at the border of the atherosclerotic lesions [36]. In patients with prehypertension and hypertension, the endothelial repair capacity of early EPCs is reduced, and likely represents an essential event in the development of hypertension [37]. Furthermore, previous studies have shown that MPs contribute to the activation of an angiogenic program in EPCs [38], MP depletion reduces the angiogenic activity of their conditioned medium [39], and *in vitro,* MPs isolated from HH hamsters reduced significantly the contractile and relaxant function of the arterial wall [5].

Thus, in the present study we focus on the effects of PBMCs-derived EPC based- therapy on platelet functions in order to explore the mechanisms that lie underneath the relationship between these cell types in vascular repair and atherosclerosis. To this purpose, the hypertensive hypercholesterolemic hamster, described previously by our group [5], was used as experimental model. Besides, we investigated contribution of PMPs in vivo, in a new animal model, HH-PMPs, and also we explored the role of PMPs on EPC actions, in another original experimental model, HH-EPCs-PMPs.

First, we investigate the EPC and PMP effects on the biochemical and hemodynamic parameters. The results showed that EPC administration (both in prevention and regression groups) has a good effect reducing the levels of serum total cholesterol, triglyceride and also, the values for systolic and diastolic blood pressure, which were significantly increased in the HH model. Moreover, it was shown that EPC therapy was more efficient in the regression group. The results obtained for HH-PMPs revealed increased levels for al tested parameters compared to HH group, indicated that PMPs accelerate the progression of atherosclerosis. The combination of EPCs with PMPs induced increased level only for cholesterol, while the triglyceride, blood pressure and heart rate were reduced compared to HH group, and also reduced compared to PMP administration, only. These experimental animal models and the effects of EPCs and PMPs on the vascular wall structure will be extensively described in a future paper.

Since platelets are considered to be essential both in atherosclerosis and in vascular and tissue regeneration through paracrine mechanisms, we focused on their relationship with EPCs. Although the effect of platelets on EPCs homing and their differentiation to endothelial cells has been well-documented, the functional consequences of these interactions on EPCs and platelets have received less attention. Moreover, we evaluated the role of PMPs, alone and in correlation with EPCs on platelets in the original experimental models.

We questioned the consequences of EPC, PMP administration (alone and in combination) on molecules involved in platelet activation (such as integrin β3), and on α_IIb_β_3_ signaling (such as FAK, PI3K and Src). Our results present a marked improvement of platelet function after EPC-based therapy in both situation (prevention and regression), compared to HH group. These findings are in concordance with the study of Abou-Saleh et al. [24] that demonstrated that *in vitro* and into mice with FeCl_3_-induced carotid artery injury EPCs bind platelets via P-selectin and inhibit platelet activation, aggregation, adhesion to collagen, and thrombus formation. The platelet activation in hypertension associated with hypercholesterolemia was revealed also in our previous study performed on HH experimental model (Alexandru et al., 2011). PMP administration enhanced platelet activation, and in combination with EPCs induced a decreased of these molecule expression compared to HH-PMPs group, but without the same results as EPC therapy.

The immediate presence of platelets at the atherosclerotic lesions renders them a potential checkpoint regulator of downstream events [40]. They can release a plethora of inflammatory mediators, enriching and boosting the inflammatory milieu. Moreover, upon activation, platelets released from the α-granules growth factors (e.g., PDGF, transforming growth factor-β, VEGF), and active metabolites that influence clinical situations requiring rapid healing and tissue regeneration [41]. Platelets chemokines (e.g. RANTES, PF4, SDF-1, MCP-1, CXCL5, CXCL7) and newly synthesized active cytokine-like factors [e.g. IL-1β, CD40 ligand (CD40L), β-thromboglobulin] are implicated in the development of atherosclerosis [41], [42], [43]. Recently, animal and (pre)clinical human studies have suggested that the two major platelet chemokines PF4 and RANTES, as well as CD40L, may be considered potential new candidates in the treatment of atherogenesis and inflammation [44]. Likewise, the SDF-1α/CXCR4 axis has been shown to be implicated in the mobilization and EPC homing [45]. Stellos et al. [23] reported that platelet-derived SDF-1α enhanced the accumulation of CD34^+^ cells at sites of injury after intravenously injection of CD34^+^ cells.

To elucidate the potential underlying mechanism involved in EPCs-platelets relationship, we compared the SDF-1α, RANTES, MCP-1 released levels, as well as their protein expression, in activated platelets isolated from hamster groups and we found an increased concentration in HH group, compared to C group and more elevated in HH-PMPs group compared to HH group. The finding of increased expression of SDF-1 in platelets from HH hamsters is consistent with the reports assessing SDF-1α in platelets from patients with cardiovascular risk factors [4] and in peripheral blood and hearts of patients with cardiovascular disease [46]. The higher values in platelets obtained from HH-PMPs group than in HH group confirmed that MPs contribute to the inflammatory milieu in atherosclerosis acting on platelet secretion. The treatment with EPCs reduced the values for these chemokines close to those in C group, suggesting that EPC-based therapy attenuated inflammation at the site of atherosclerotic lesion in hypertension associated with hypercholesterolemia. The simultaneous administration of EPCs and PMPs, for the first time used in atherosclerosis, induced a decrease of the protein expression and a release of the investigated chemokines compared to HH-PMPs group, but without the same efficiency as in EPC groups.

For another investigated factor, PF4, we obtained also the higher concentrations in platelet supernatants obtained from HH and HH-PMPs groups, suggesting the implication of this molecule in atherosclerosis. Our results are in agreement with a previous study in that was demonstrated that platelet PF4 promotes atherosclerotic lesion development in vivo [47]. Moreover, it was showed that PF4 plays a pro-atherogenic role by interacting with the vascular endothelium and monocytes, facilitating monocyte differentiation to inflammatory macrophages and monocyte recruitment to the arterial wall during atherosclerotic plaque development and by increasing the uptake and esterification of ox-LDL in lesional macrophages [48]. In addition, the presence of PF4 and RANTES together is associated with the progression of atherosclerosis [42] and is involved in the vascular remodeling after injury [31]. The PF4- RANTES heterodimers have been proposed to represent potential therapeutic targets in the treatment of atherosclerosis [49]. The EPC-therapy reduced significantly the levels of PF4 and in addition has a good result in correlation with PMPs.

Platelets store and release also a number of regulators that modulate angiogenesis [23], [50] and likewise, PMPs are potent inducers of angiogenesis [51]. The levels of pro-angiogenic factors, including VEGF, PDGF and cytokines: IL-1β, Il-6 were significantly increased in platelets obtained from HH and HH-PMPs groups, and are correlated with the results got in platelets from patients with cardiovascular risk factors [4]. Analysis of platelets from HH-EPCs groups showed a reduction of these protein expression at values closed to C group suggesting the efficiency of EPC therapy, while the combination between EPCs and PMPs reduced the values compared to HH-PMPs group, but being useful only on diminishing VEGF and IL-6 expressions to the values compared to C group.

Platelets activation and granule release play a crucial role in both atherogenesis and acute atherothrombosis. One of the early events after vascular disruption, and complementary to platelet activation, is the activation of coagulation cascade [32]. Since, recently it was shown that TFPI present within platelets functions to limit intravascular thrombus growth, likely through the inhibition of procoagulant activity of blood borne TF [52]. In our study we determinated the concentration of this factor in platelet supernatant released by activated platelets. Compared to C group, we obtained significantly decreased levels of TFPI in platelets from HH and HH-PMPs groups, and similar values for platelets from the groups that have been treated with EPCs. These results confirm the beneficial effects of these cells in the improvement of platelet functions.

Taken together, the data obtained in the present study on the new animal models underline that circulating EPC-based therapy: (i) have the ability to improve plasmatic and hemodynamic parameters; (ii) reduced platelet activation and modulated their pro-inflammatory and thrombogenic properties during atherosclerotic process; (iii) in combination with PMPs have several beneficial effects on platelet function, but no better than in situation without microparticles. Although, some studies reported that PMPs enhance the potential of EPCs to restore endothelial integrity after vascular injury [53], while PMP injections were sufficient to stimulate postischemic revascularization in the myocardium, in a rat model of chronic myocardial ischemia [54], our results suggested that PMPs did not improve the EPC effects on the platelet activation induced by hypertension associated with hypercholesterolemia.

This study reveals a new biological role for circulating EPCs in regulation of platelet function in atherosclerosis. However, the existence of a cross talk between EPCs and platelets, in each other’s function regulation, requires future studies to explore the interactions between these cells and the mechanisms that underlie this relationship in vascular repair and atherosclerosis.

## References

[1] Schmidt-Lucke C, Rössig L, Fichtlscherer S, Vasa M, Britten M, et al (2005) Reduced number of circulating endothelial progenitor cells predicts future cardiovascular events: proof of concept for the clinical importance of endogenous vascular repair. Circulation 111: 2981–2987. 15927972 10.1161/CIRCULATIONAHA.104.504340

[2] Heiss C, Keymel S, Niesler U, Ziemann J, Kelm M, et al (2005) Impaired progenitor cell activity in age-related endothelial dysfunction. J Am Col. Cardiol 45: 1441–1448. 10.1016/j.jacc.2004.12.074 15862416

[3] Urbich C, Dimmeler S (2005) Risk factors for coronary artery disease, circulating endothelial progenitor cells, and the role of HMG-CoA reductase inhibitors. Kidney Int 67: 1672–1676. 15840010 10.1111/j.1523-1755.2005.00261.x

[4] Dernbach E, Randriamboavonjy V, Fleming I, Zeiher AM, Dimmeler S, et al (2008) Impaired interaction of platelets with endothelial progenitor cells in patients with cardiovascular risk factors. Basic Res Cardiol 103: 572–581. 18604625 10.1007/s00395-008-0734-z

[5] Georgescu A, Alexandru N, Andrei E, Titorencu I, Dragan E, et al (2012) Circulating microparticles and endothelial progenitor cells in atherosclerosis; pharmacological effects of irbesartan. J Thromb Haemost 10(4): 680–91. 22303879 10.1111/j.1538-7836.2012.04650.x

[6] Xu Q (2007) Progenitor cells in vascular repair. Curr Opin Lipidol 18: 534–9. 17885424 10.1097/MOL.0b013e3282a66082

[7] Fadini GP, Avogaro A (2010) Cell-based methods for ex vivo evaluation of human endothelial biology. Cardiovascular Research 87: 12–21. 20427336 10.1093/cvr/cvq119

[8] Werner N, Nickenig G (2006) Clinical and therapeutical implications of EPC biology in atherosclerosis. J Cell Mol Med 10: 318–332. 16796802 10.1111/j.1582-4934.2006.tb00402.x PMC3933124

[9] Yoder MC, Mead LE, Prater D, Krier TR, Mroueh KN, et al (2007) Redefining endothelial progenitor cells via clonal analysis and hematopoietic stem/progenitor cell principals. Blood 109: 1801–1809. 17053059 10.1182/blood-2006-08-043471 PMC1801067

[10] Co M, Tay E, Lee CH, Poh KK, Low A, et al (2008) Use of endothelial progenitor cell capture stent (Genous Bio-Engineered R Stent) during primary percutaneous coronary intervention in acute myocardial infarction: intermediate- to long-term clinical follow-up. Am Heart J 155: 128–132. 18082503 10.1016/j.ahj.2007.08.031

[11] Miglionico M, Patti G, D’Ambrosio A, Di Sciascio G (2008) Percutaneous coronary intervention utilizing a new endothelial progenitor cells antibody-coated stent: a prospective single-center registry in high-risk patients. Catheter Cardiovasc Interv 71: 600–604. 18360849 10.1002/ccd.21437

[12] Larsen K, Cheng C, Tempel D, Parker S, Yazdani S, et al (2012) Capture of circulatory endothelial progenitor cells and accelerated re-endothelialization of a bio-engineered stent in human ex vivo shunt and rabbit denudation model. Eur Heart J 33(1): 120–128. 21733913 10.1093/eurheartj/ehr196 PMC3249218

[13] Davi G, Patrono C (2007) Platelet activation and atherothrombosis. N Engl J Med 357: 2482–2494. 18077812 10.1056/NEJMra071014

[14] Stellos K, Gawaz M (2007) Platelet interaction with progenitor cells: potential implications for regenerative medicine. Thromb Haemost 98: 922–929. 18000594 10.1160/th07-02-0147

[15] Lindemann S, Kraemer B, Daub K, Stellos K, Gawaz M (2007) Molecular pathways used by platelets to initiate and accelerate atherogenesis. Curr Opin Lipidol 8: 566–573. 10.1097/MOL.0b013e3282ef7c1e 17885429

[16] Tsoumani ME, Kalantzi KI, Goudevenos IA, Tselepis AD (2012) Platelet-mediated inflammation in cardiovascular disease. Potential role of platelet-endothelium interactions. Curr Vasc Pharmacol 10(5): 539–49. 22338568 10.2174/157016112801784602

[17] Lev EI, Estrov Z, Aboulfatova K, Harris D, Granada JF, et al (2006) Potential role of activated platelets in homing of human endothelial progenitor cells to subendothelial matrix. Thromb Haemost 96: 498–504. 17003929

[18] Langer H, May AE, Daub K, Heinzmann U, Lang P, et al (2006) Adherent platelets recruit and induce differentiation of murine embryonic endothelial progenitor cells to mature endothelial cells in vitro. Circ Res 98(2): e2–10. 16373597 10.1161/01.RES.0000201285.87524.9e

[19] Daub K, Langer H, Seizer P, Stellos K, May AE, et al (2006) Platelets induce differentiation of human CD34+ progenitor cells into foam cells and endothelial cells. Faseb J 20: 2559–2561. 17077283 10.1096/fj.06-6265fje

[20] Hristov M, Zernecke A, Bidzhekov K, Liehn EA, Shagdarsuren E, et al (2007) Importance of CXC chemokine receptor 2 in the homing of human peripheral blood endothelial progenitor cells to sites of arterial injury. Circ Res 100: 590–597. 17272812 10.1161/01.RES.0000259043.42571.68

[21] Massberg S, Konrad I, Schurzinger K, Lorenz M, Schneider S, et al (2006) Platelets secrete stromal cellderived factor 1alpha and recruit bone marrow-derived progenitor cells to arterial thrombi in vivo. J Exp Med 203: 1221–1233. 16618794 10.1084/jem.20051772 PMC2121205

[22] Leshem-Lev D, Omelchenko A, Perl L, Kornowski R, Battler A, et al (2010) Exposure to platelets promotes functional properties of endothelial progenitor cells. J Thromb Thrombolysis 30: 398–403. 20734120 10.1007/s11239-010-0514-0

[23] Stellos K, Langer H, Daub K, Schoenberger T, Gauss A, et al (2008) Platelet-derived stromal cell-derived factor-1 regulates adhesion and promotes differentiation of human CD34+ cells to endothelial progenitor cells. Circulation 117(2): 206–215. 18086932 10.1161/CIRCULATIONAHA.107.714691

[24] Abou-Saleh H, Yacoub D, Théorêt J-F, Gillis MA, Neagoe PE, et al (2009) Endothelial Progenitor Cells Bind and Inhibit Platelet Function and Thrombus Formation. Circulation 120: 2230–2239. 19917882 10.1161/CIRCULATIONAHA.109.894642 PMC3694872

[25] Flaumenhaft R (2006) Formation and fate of platelet microparticles. Blood Cells, Molecules, and Diseases 36: 182–187. 10.1016/j.bcmd.2005.12.019 16466949

[26] Georgescu A, Alexandru N, Popov D, Amuzescu M, Andrei E, et al (2009) Chronic venous insufficiency is associated with elevated level of circulating microparticles. J Thromb Haemost 7(9): 1566–1575. 19552639 10.1111/j.1538-7836.2009.03525.x

[27] Alexandru N, Popov D, Dragan E, Andrei E, Georgescu A (2011) Platelet activation in hypertension associated with hypercholesterolemia: effects of irbesartan. J Thromb Haemost 9(1): 173–84. 20961397 10.1111/j.1538-7836.2010.04122.x

[28] He T, Smith LA, Harrington S, Nath KA, Caplice NM, et al (2004) Transplantation of Circulating Endothelial Progenitor Cells Restores Endothelial Function of Denuded Rabbit Carotid Arteries. Stroke 35: 2378–2384. 15345801 10.1161/01.STR.0000141893.33677.5d

[29] Lupu C, Calb M, Ionescu M, Lupu F (1993) Enhanced prothrombin and intrinsic factor X activation on blood platelets from diabetic patients. Thromb Haemost 70: 579–83. 8115982

[30] Bergmeier W, Schulte V, Brockhoff G, Bier U, Zirngibl H, et al (2002) Flow cytometric detection of activated mouse integrin alphaIIbbeta3 with a novel monoclonal antibody. Cytometry 48: 80–6. 12116368 10.1002/cyto.10114

[31] Kaplan ZS, Jackson SP (2011) The Role of Platelets in Atherothrombosis. Hematology Am Soc Hematol Educ Program 2011: 51–61. 22160012 10.1182/asheducation-2011.1.51

[32] Badimon L, Padró T, Vilahur G (2012) Atherosclerosis, platelets and thrombosis in acute ischaemic heart disease. European Heart Journal: Acute Cardiovascular Care 1: 60–74. 24062891 10.1177/2048872612441582 PMC3760546

[33] Georgescu A (2011) Vascular dysfunction in diabetes: The endothelial progenitor cells as new therapeutic strategy. World J Diabetes 2(6): 92–97. 21860692 10.4239/wjd.v2.i6.92 PMC3158877

[34] Foteinos G, Hu Y, Xiao Q Metzler B, Xu Q (2008) Rapid endothelial turnover in atherosclerosis-prone areas coincides with stem cell repair in apolipoprotein E-deficient mice. Circulation 117: 1856–1863. 18378610 10.1161/CIRCULATIONAHA.107.746008

[35] Xu Q, Zhang Z, Davison F, Hu Y (2003) Circulating progenitor cells regenerate endothelium of vein graft atherosclerosis, which is diminished in ApoE deficient mice. Circ Res 93: e76–e86. 14512446 10.1161/01.RES.0000097864.24725.60

[36] Ii M, Takeshita K, Ibusuki K, Luedemann C, Wecker A, et al (2010) Notch signaling regulates endothelial progenitor cell activity during recovery from arterial injury in hiypercholesterolemic mice. Circulation 121: 1104–1112. 20176991 10.1161/CIRCULATIONAHA.105.553917 PMC2838724

[37] Giannotti G, Doerries C, Mocharla PS, Mueller MF, Bahlmann FH, et al (2010) Impaired Endothelial Repair Capacity of Early Endothelial Progenitor Cells in Prehypertension: Relation to Endothelial Dysfunction. Hypertension 55: 1389–1397. 20458006 10.1161/HYPERTENSIONAHA.109.141614

[38] Deregibus MC, Cantaluppi V, Calogero R, Lo Iacono M, Tetta C, et al (2007) Endothelial progenitor cell derived microvesicles activate an angiogenic program in endothelial cells by a horizontal transfer of mRNA. Blood 110: 2440–2448. 17536014 10.1182/blood-2007-03-078709

[39] Prokopi M, Pula G, Mayr U, Devue C, Gallagher J, et al (2009) Proteomic analysis reveals presence of platelet microparticles in endothelial progenitor cell cultures. Blood 114: 723–732. 19369228 10.1182/blood-2009-02-205930

[40] Langer HF, Gawaz M (2008) Platelets in regenerative medicine. Basic Res Cardiol 103(4): 299–307. 18392766 10.1007/s00395-008-0721-4

[41] Gawaz M, Langer H, May AE (2005) Platelets in inflammation and atherogenesis. J Clin Invest 115: 3378–3384. 16322783 10.1172/JCI27196 PMC1297269

[42] McNicol A, Israels SJ (2008) Beyond Hemostasis: The Role of Platelets in Inflammation, Malignancy and Infection. Cardiovascular & Haematological Disorders-Drug Targets 8: 99–117. 18537597 10.2174/187152908784533739

[43] Lievens D, von Hundelshausen P (2011) Platelets in atherosclerosis. Thromb Haemost 106: 827–838. 22012554 10.1160/TH11-08-0592

[44] Nagy B, Miszti-Blasiushtt K, Kerenyi A, Clemetson KJ, Kappelmayer J (2012) Potential therapeutic targeting of platelet-mediated cellular interactions in atherosclerosis and inflammation. Curr Med Chem 19(4): 518–31. 22204330 10.2174/092986712798918770

[45] Walter DH, Haendeler J, Reinhold J, Rochwalsky U, Seeger F, et al (2005) Impaired CXCR4 signaling contributes to the reduced neovascularization capacity of endothelial progenitor cells from patients with coronary artery disease. CircRes 97: 1142–1151. 10.1161/01.RES.0000193596.94936.2c 16254213

[46] Theiss HD, David R, Engelmann MG, Barth A, Schotten K, et al (2007) Circulation of CD34+ progenitor cell populations in patients with idiopathic dilated and ischaemic cardiomyopathy (DCM and ICM). Eur Heart J 28: 1258–1264. 17395679 10.1093/eurheartj/ehm011

[47] Sachais BS, Turrentine T, Dawicki McKenna JM, Rux AH, Rader D, et al (2007) Elimination of platelet factor 4 (PF4) from platelets reduces atherosclerosis in C57Bl/6 and apoE−/− mice. Thromb Haemost 98(5): 1108–13. 18000617

[48] Aidoudi S, Bikfalvi A (2010) Interaction of PF4 (CXCL4) with the vasculature: A role in atherosclerosis and angiogenesis. Thromb Haemost 104: 941–948. 20806113 10.1160/TH10-03-0193

[49] Koenen RR, von Hundelshausen P, Nesmelova IV, Zernecke A, Liehn EA, et al (2009) Disrupting functional interactions between platelet chemokines inhibits atherosclerosis in hyperlipidemic mice. Nature medicine 15(1): 97–103. 10.1038/nm.1898 19122657

[50] Gawaz M, Stellos K, Langer HF (2008) Platelets modulate atherogenesis and progression of atherosclerotic plaques via interaction with progenitor and dendritic cells. J Thromb Haemost 6(2): 235–242. 18088342 10.1111/j.1538-7836.2008.02867.x

[51] Boulanger CM, Tedgui A (2005) Dying for attention: microparticles and angiogenesis. Cardiovasc Res 67: 1–3. 15904903 10.1016/j.cardiores.2005.05.001

[52] Maroney SA, Cooley BC, Ferrel JP, Bonesho CE, Mast AE (2011) Murine hematopoietic cell tissue factor pathway inhibitor limits thrombus growth.Arterioscler Thromb Vasc Biol. 31(4): 821–6. 10.1161/ATVBAHA.110.220293 PMC3060281 21233452

[53] Mause SF, Ritzel E, Liehn EA, Hristov M, Bidzhekov K, et al (2010) Platelet microparticles enhance the vasoregenerative potential of angiogenic early outgrowth cells after vascular injury. Circulation 122: 495–506. 20644015 10.1161/CIRCULATIONAHA.109.909473

[54] Brill A, Dashevsky O, Rivo J, Gozal Y, Varon D (2005) Platelet-derived microparticles induce angiogenesis and stimulate post-ischemic revascularization. Cardiovasc Res 67: 30–38. 15878159 10.1016/j.cardiores.2005.04.007

